# Supplementary material for: Synthesis and Characterization of Cu2ZnSnSe4 by Non-Vacuum Method for Photovoltaic Applications
Source: Nanomaterials (Basel). 2022 Apr 28;12(9):1503. doi: 10.3390/nano12091503 (PMC9100807; doi:10.3390/nano12091503)
Supplement: Supplementary file 1 [file nanomaterials-12-01503-s001.zip › nanomaterials-1673580-supplementary.pdf]

Electronic Supplementary Information

Synthesis and Characterization of $Cu_2ZnSnSe_4$ by Non-Vacuum Method for Photovoltaic Applications

Meenakshi Sahu [1,2], Vasudeva Reddy Minnam Reddy [3], Bharati Patro [4], Chinho Park [2,*], Woo Kyoung Kim [3,*] and Pratibha Sharma [1,*]

1 Department of Energy Science and Engineering, Indian Institute of Technology Bombay Powai, Mumbai 400076, India; meenakshisahu.chem@gmail.com
2 Korea Institute of Energy Technology (KENTECH), 200 Hyukshin-ro, Naju 58330, Korea
3 School of Chemical Engineering, Yeungnam University, Gyeongsan 38541, Korea; drmvasudr9@gmail.com
4 Centre for Research in Nanotechnology and Sciences, Indian Institute of Technology Bombay Powai, Mumbai 400076, India; bharati@iitb.ac.in
* Correspondence: chpark@kentech.ac.kr (C.P.); wkim@ynu.ac.kr (W.K.K.); pratibha_sharma@iitb.ac.in (P.S.)

Content	Page No
• Title page	**S1**
• Preparation of CZTSe nanopowder, ink and sodium solution	**S2**
• CZTSe thin-film coating, annealing, and photovoltaic device fabrication	**S2**
• Figure S1: Green synthesis of CZTSe nanoparticle and ink formation by wet ball milling method	**S2**
• Characterization	**S3**
• Table S1: Structural parameter: crystalline (D), strain (ε), dislocation density(δ) and lattice constant (a=b and c) of CZTSe thin film annealed at different temperatures	**S4**
• Table S2: Relative intensity ratio of CZTSe (112), (204) and (312) plane	**S4**
• Table S3: Raman scattering band position of CZTSe and other secondary phases	**S5**
• Figure S2: Surface and cross-section images and EDS spectra of Se0 thin film	**S5**
• Figure S3: Surface and cross-section images and EDS of Se0_Na thin film with sodium layer	**S6**
• Figure S4: Surface and cross-section images and EDS spectra of Se3 thin film	**S6**
• Figure S5: Surface and cross-section images and EDS spectra of Se3_Na thin film with sodium layer	**S7**
• Table S4: Elemental composition of CZTSe without sodium layer	**S7**
• Table S5: Elemental composition of CZTSe with sodium layer	**S7**
• References	**S8**

Preparation of CZTSe nanopowder, ink, and sodium solution

CZTSe nanopowder, ink, and sodium solution were prepared similar to our previously reported study. CZTSe nanopowder and ink were synthesized by the wet ball milling process and detailed process are given in the previous study [23]. Elemental powders of Cu, Zn, Sn and Se, and butanol were fed into a ball milling jar and milled at 450 rpm for 30 hours (Figure S1). For ink preparation, the CZTSe nanopowders, azeotropic solvent (a mixture of ethanol and MEK), binder, and surfactant (Tween-80 and PEG-400) were added into a vial and milled for > 48 hours to prepare a stable ink. The ink prepared was named as "***CZTSe-Ink***" in this manuscript (Figure S1). For sodium solution, 1.0 mmol of selenium powder added into $NaBH_4$ (1.2 mmol)/C_2H_5OH (25 ml) solution and stirred for 15 min at 75 °C.

CZTSe thin-film coating, annealing, and photovoltaic device fabrication

CZTSe-based thin films and photovoltaic device with and without sodium-containing were fabricated analogous to previously reported work [23]. Formulated CZTSe inks were used to deposit thin films by a spin coating method. The coating process was repeated three times, and each layer of the thin film was pre-heated (air at 75 °C for 1 min) to obtain the required thickness. For fabrication of sodium-containing samples, a two-layer of prepared sodium solution was spin-coated on the top of the CZTSe thin film. For annealing process, thin films were loaded in a graphite box with selenium powder and annealed at 500 °C, 520 °C and 550 °C for 30 min in rapid thermal processing (RTP) system under a continuous flow of N_2 gas. The fabricated CZTSe thin films were integrated into photovoltaic devices in the traditional configuration of Mo/CZTSe/CdS/ZnO/AZO/Ni/Al without sodium, and Mo/CZTSe:Na/CdS/ZnO/AZO/Ni/Al with sodium. Other layers such as buffer layer, window layers and metal grid was deposited analogous to the standard process reported in the literature [23].

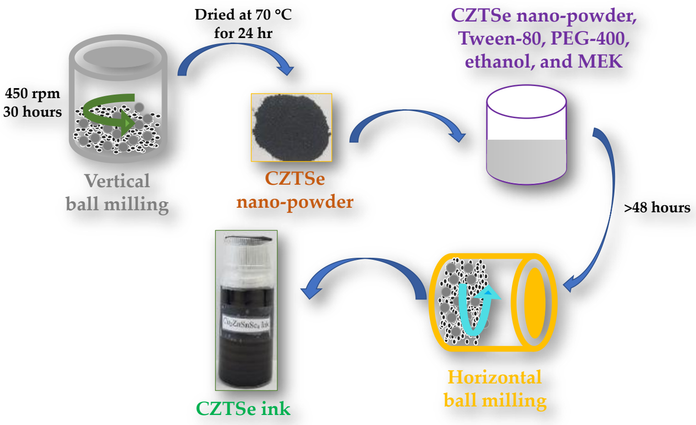

Figure S1. Green synthesis of CZTSe nanoparticle and ink formation by wet ball milling method

Characterization

Various characterization techniques such as zeta potential, dynamic light scattering (DLS), XRD, Raman spectrometer, FE-SEM, (EDS), Fourier transform infrared spectroscopy (FT-IR), UV-Vis-NIR spectrometer and Hall measurement were used to analyze the prepared CZTSe samples. The ink stability and hydrodynamic size of CZTSe nanoparticles were measured using Malvern Zetasizer Nano ZS dynamic light scattering (Worcestershire, UK). The crystallographic information of the before- and after-annealed thin films was identified by an X-ray diffractometer (Grazing-incidence XRD and Bulk-XRD: PANalytical X'Pert-PRO MPD, Almelo, The Netherlands) using the monochromatized Cu-Kα radiation (λ = 1.5406 Å), a grazing angle of 1.0° (for Thin film-XRD), 2θ range from 10° to 80° at a scan rate of 4°/min and step size of 0.02°. Phase analysis of CZTSe samples was performed using Jobin-Yvon-Horiba (model no-HR800UV, Horiba Jobin Yvon, France) Raman spectrometer at room temperature with a 514.5 nm wavelength laser and working power at 10 mW. The surface morphology and cross-sectional images of the samples were observed using FE-SEM (Hitachi, model no. S-4800, Cary 5000, Agilent, Santa Clara, CA, USA). The EDS was used to estimate the chemical composition of the samples. The optical properties of all the CZTSe thin films were measured in the wavelength range of 300 – 2500 nm using a UV-Vis-NIR spectrometer (Varian, model no-UV-5000, Tokyo, Japan). The electrical properties were measured by using the Hall effect measurement (model no. HMS5000, Gyeonggi, Korea) with the Van der Paw approach with the current in the range of 1.0 – 0.5 μA and magnetic field intensity of 0.55 Tesla at room temperature. The silver paste was used to make metal contact on the corner of the thin film. Fourier transform infrared spectra were collected using Perkin Elmer FT-IR Spectrometer (model no. Spectrum-100, Thermo Scientific; Waltham, MA, USA) in KBr mode. The current density-voltage (J-V) curve of the fabricated photovoltaic device with approximately 0.4 × 0.4 cm^2 area was measured with an AAA-class solar simulator (K201, LAB 55, McScience, Korea) and Keithley (model no. 2400) as a Source Meter SMU Instrument. A Xenon flash lamp (USHIO, flash type, UA-DF1, 1,000 W, 400 V) with a standard AM1.5 (100 mW/cm^2) filter was used as a source of light.

Table S1. Structural parameter: crystalline (D), strain (ε), dislocation density(δ) and lattice constant (a=b and c) of CZTSe thin film annealed at different temperature

**Parameter**		**Sample Name**				
		**CZTSe As Synthesized**	**CZTSe (Se0)**	**CZTSe-500 (Se1)**	**CZTSe-520 (Se2)**	**CZTSe-550 (Se3)**
Phase		**Kesterite**				
Scherrer	***D* (nm)**	**9.47**	**6.97**	**45.00**	**36.13**	**21.63**
	***δ* (nm^{-2})**	**0.01114**	**0.02057**	**0.00049**	**0.00076**	**0.00213**
Williamson-Hall	***D* (nm)**	**7.21**	**7.53**	**50.41**	**38.73**	**19.63**
	***ε* (× 10^{-3})**	**−4.5**	**−6.7**	**−0.06**	**−0.1**	**−1.0**
	***δ* (nm^{-2})**	**0.01923**	**0.01759**	**0.00039**	**0.00066**	**0.00259**
Lattice Constant	***a* (Å)**	**5.67**	**5.66**	**5.64**	**5.65**	**5.66**
	***c* (Å)**	**11.34**	**11.34**	**11.30**	**11.33**	**11.36**
	c*/2*a	**1.00**	**1.00**	**1.00**	**1.00**	**1.00**
Lattice Structure		**Tetragonal**				

Table S2. Relative intensity ratio of CZTSe (112), (204) and (312) plane

**Sample Name**	$I_{(112)}/I_{(204)}$	$I_{(112)}/I_{(312)}$	$I_{(112)}/I_{(204)}$	$I_{(112)}/I_{(312)}$
**Se0**	**2.21**	**4.51**		
**Se1**	**3.77**	**11.77**		
**Se2**	**2.19**	**5.16**		
**Se3**	**1.75**	**3.58**	**2.63**	**5.0**
**Se0_Na**	**2.37**	**4.24**		
**Se1_Na**	**2.94**	**7.86**		
**Se2_Na**	**1.84**	**3.97**		
**Se3_Na**	**1.71**	**3.43**		

Table S3. Raman scattering band position of CZTSe and other secondary phases

Sl. No.	Phase	Raman peak position (cm^{-1})	Ref.
1	$Cu_2ZnSnSe_4$	173, 196, and 231	[59,60]
2	ZnSe	200 250	[60]
6	Monoclinic Cu_2SnSe_3	83, 180, 236, 251	[59]
8	Cubic Cu_2SnSe_3	77.8, 179.9	[61]
9	$Cu_{2-x}Se$	262	[62]
11	$SnSe_2$	107.3, 118.2, 185.4	[61]
12	SnSe	70.1, 109, 130.6, 150, 184.5	[62]

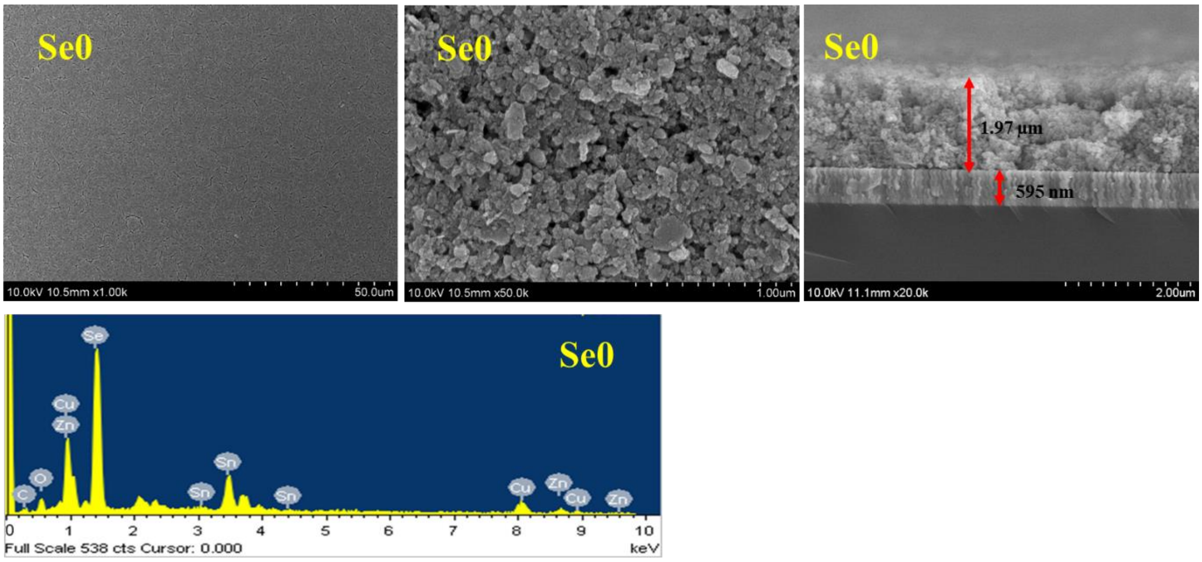

Figure S2. Surface and cross-section images and EDS spectra of Se0 thin film

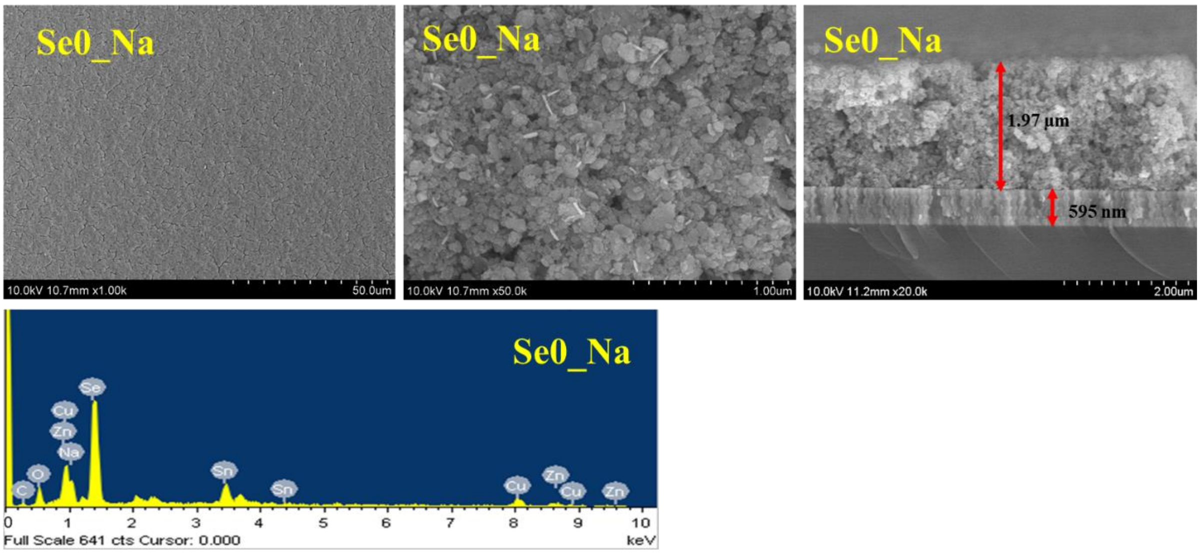

Figure S3. Surface and cross-section images and EDS of Se0_Na thin film with sodium layer

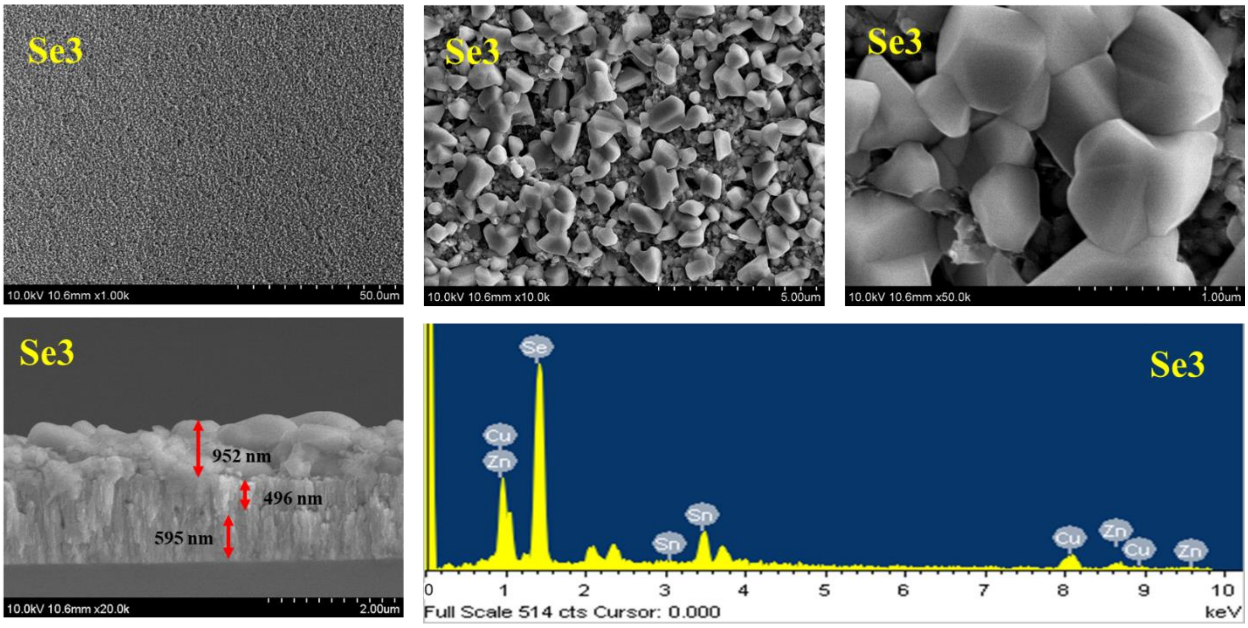

Figure S4. Surface and cross-section images and EDS spectra of Se3 thin film

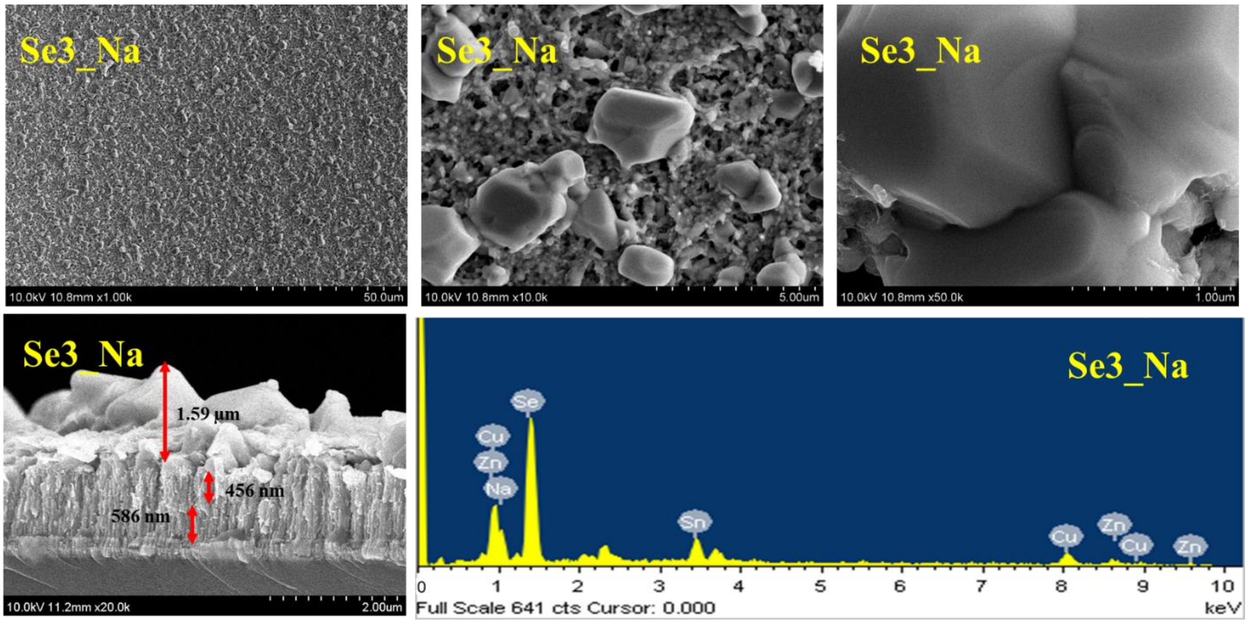

Figure S5. Surface and cross-section images and EDS spectra of S3_Na thin film with sodium layer

Table S4. Atomic percentage of CZTSe without sodium layer

Sl No	Sample Name	Cu%	Zn%	Sn%	Se%	C%	O%
1	Se0	17.37	8.49	7.92	29.10	15.46	21.66
2	Se1	26.25	14.99	12.54	46.21	-	-
3	Se2	27.37	12.83	12.56	47.24	-	-
4	Se3	24.75	15.51	11.67	48.07	-	-

Table S5. Atomic percentage of CZTSe with sodium layer

Sl No	Sample Name	Cu%	Zn%	Sn%	Se%	Na%	C%	O%
1	Se0_Na	13.18	5.28	6.42	25.65	4.90	16.65	27.92
2	Se1_Na	27.58	10.25	12.25	44.79	5.14	-	-
3	Se2_Na	26.11	9.83	12.46	44.81	6.80	-	-
4	Se3_Na	25.23	11.51	10.88	47.03	5.35	-	-

References

23. Sahu, M.; Reddy, V.R.M.; Kim, B.; Patro, B.; Park, C.; Kim, W.K.; Sharma, P. Fabrication of Cu2ZnSnS4 Light Absorber Using a Cost-Effective Mechanochemical Method for Photovoltaic Applications. Materials **2022**, *15*, 1708.

59. Altosaar, M.; Raudoja, J.; Timmo, K.; Danilson, M.; Grossberg, M.; Krustok, J.; Mellikov, E. $Cu_2Zn_{1-x}Cd_xSn(Se_{1-y}S_y)_4$ solid solutions as absorber materials for solar cells. *Phys. Status Solidi (A)* **2008**, *205*, 167–170.

60. Redinger, A.; Hönes, K.; Fontané, X.; Izquierdo-Roca, V.; Saucedo, E.; Valle, N.; Pérez-Rodríguez, A.; Siebentritt, S. Detection of a ZnSe secondary phase in coevaporated $Cu_2ZnSnSe_4$ thin films. *Appl. Phys.Lett.* **2011**, *98*, 101907.

61. Kim, Y.; Choi, I.-H. High pressure Raman scattering of a co-evaporated Cu_2SnSe_3 thin film. *Thin SolidFilm.* **2018**, *647*, 9–12.

62. Chihi, A.; Bessais, B. Synthesis and characterization of Cu_2SnSe_3 thin films by electrodeposition route. *SuperlatticesMicrostruct.* **2016**, *97*, 287–297.
